# Hsp-27 expression at diagnosis predicts poor clinical outcome in prostate cancer independent of ETS-gene rearrangement

**DOI:** 10.1038/sj.bjc.6605227

**Published:** 2009-08-25

**Authors:** C S Foster, A R Dodson, L Ambroisine, G Fisher, H Møller, J Clark, G Attard, J De-Bono, P Scardino, V E Reuter, C S Cooper, D M Berney, J Cuzick

**Affiliations:** 1 Division of Cellular Pathology and Molecular Genetics, University of Liverpool, Duncan Building, Daulby Street, Liverpool L69 3GA, UK; 2 Cancer Research UK Centre for Epidemiology, Mathematics and Statistics Wolfson Institute of Preventive Medicine, Queen Mary University of London, Charterhouse Square, London EC1M 6BQ, UK; 3 King's College London, Thames Cancer Registry, 42 Weston Street, London SE1 3QD, UK; 4 Institute of Cancer Research, Male Urological Cancer Research Centre, 15 Cotswold Road, Belmont, Sutton, Surrey SM2 5NG, UK; 5 Departments of Urology and Pathology, Memorial Sloan Kettering Cancer Center, 1275 York Avenue, New York, NY 10021, USA; 6 The Orchid Tissue Laboratory, Centre for Molecular Oncology, Barts and The London School of Medicine and Dentistry, London E1 2AD, UK

**Keywords:** heat shock protein 27, prostate cancer, prognostic biomarker

## Abstract

**Background:**

This study was performed to test the hypothesis that expression of small heat shock protein Hsp-27 is, at diagnosis, a reliable predictive biomarker of clinically aggressive prostate cancer.

**Methods:**

A panel of tissue microarrays constructed from a well-characterised cohort of 553 men with conservatively managed prostate cancer was stained immunohistochemically to detect Hsp-27 protein. Hsp-27 expression was compared with a series of pathological and clinical parameters, including outcome.

**Results:**

Hsp-27 staining was indicative of higher Gleason score (*P*<0.001). In tissue cores having a Gleason score >7, the presence of Hsp-27 retained its power to independently predict poor clinical outcome (*P*<0.002). Higher levels of Hsp-27 staining were almost entirely restricted to cancers lacking ERG rearrangements (*χ*^2^ trend=31.4, *P*<0.001), although this distribution did not have prognostic significance.

**Interpretation:**

This study has confirmed that, in prostate cancers managed conservatively over a period of more than 15 years, expression of Hsp-27 is an accurate and independent predictive biomarker of aggressive disease with poor clinical outcome (*P*<0.001). These findings suggest that apoptotic and cell-migration pathways modulated by Hsp-27 may contain targets susceptible to the development of biologically appropriate chemotherapeutic agents that are likely to prove effective in treating aggressive prostate cancers.

Biologically and behaviourally, prostate cancer is a heterogeneous malignant disease (Foster *et al*, 2002). In the USA, in 2008, there were 186,320 new cases of prostate cancer and 28 660 deaths from this disease (Jemal *et al*, 2008). Equivalent figures from the United Kingdom reveal that 34,302 new cases were diagnosed in 2005 and that 10 000 men died of prostate cancer (http://info.cancerresearchuk.org/cancerstats/types/prostate/). Globally, prostate cancer is currently the fifth commonest malignancy and the most common in men, with more than 679 000 new cases estimated to have occurred in 2002 (Parkin *et al*, 2005). The clinical potential of an individual prostate cancer may range from relative indolence to highly aggressive, with progression occurring rapidly. Hence, there is an urgent requirement to develop reliable biomarkers that can accurately stratify prostate cancer at diagnosis and segregate men with aggressive cancers requiring urgent treatment from those who may be managed conservatively. Thus far, the only two parameters generally accepted to be valuable in the clinical management of prostate cancer are serum PSA and Gleason score following histopathological assessment of prostatic biopsy specimens. Although the extent of disease is more useful than clinical stage at diagnosis, and retains a low level of significance in multivariate analysis, neither of these are of clinical utility and are significantly inferior to Gleason score and PSA (Cuzick *et al*, 2006). Although both approaches have been clinically valuable, neither provides accurate predictive information with respect to individual prostate cancers.

Hsp-27 was originally discovered as an oestrogen-modulated protein in breast cancer (Adams *et al*, 1983), and thereafter identified to contribute to apoptosis (Tenniswood *et al*, 1992) and as a potential prognostic marker in prostate cancer cells (Morino *et al*, 1997). The functional relationship between Hsp-27, encoded by the gene *HSPB1* located on human chromosome 7 at q11.23, and behavioural phenotype was suggested in the preliminary study that showed Hsp-27 expression to predict (*P*<0.0001) death from prostate cancer (Cornford *et al*, 2000). However, the cohort was heterogeneous and the sample size relatively small. In contrast, the current study has allowed a rigorous assessment of Hsp-27 expression in a large cohort of well-characterised and conservatively managed patients (Cuzick *et al*, 2006) with respect to the prediction of tumour aggression. Our original observation that Hsp-27 predicts clinical recurrence in prostate cancer was supported by the finding from a subsequent study that the protein level increases after androgen ablation and is cytoprotective in hormone-refractory prostate cancer (Rocchi *et al*, 2004), and, more recently, by two studies on men undergoing radical prostatectomy (Glaessgen *et al*, 2008; Miyake *et al*, 2008).

The value of Hsp-27 expression as a predictive biomarker is not restricted to prostate cancer. In developing breast neoplasia, modulation of Hsp-27 in early proliferative lesions (O'Neill *et al*, 2003) occurs synchronously with that of ER-alpha to predict subsequent development of breast malignancy (*P*<0.001). However, in established node-positive breast cancer, Hsp-27 does not predict survival (Tetu *et al*, 1995). In cervical neoplasia, association has been reported between over-expression of Hsp-27 and grade, although no survival data were included (Ono *et al*, 2009). With respect to treatment of malignant disease, increased expression of Hsp-27 has been linked to vincristine resistance in gastric cancer (Yang *et al*, 2009) and to 5-fluorouracil resistance in colon cancer (Tsuruta *et al*, 2008), and breast cancer cells initially over-expressing Hsp-27 became sensitive to doxorubicin after modulation of endogenous Hsp-27 levels by paclitaxel (Shi *et al*, 2008).

Prostate cancers often contain an *ETS-*gene fusion that involves the androgen-regulated promoter regions from the *TMPRSS* gene (chromosome 21q22.3) and joins to 3′ *ERG* sequences (Tomlins *et al*, 2005). These translocations are common in established prostate cancer (Clark *et al*, 2007, 2008) and have been identified in PIN, but with a lower frequency (Cerveira *et al*, 2006; Perner *et al*, 2007; Mosquera *et al*, 2008). Taken together with evidence from transgenic mouse studies (Tomlins *et al*, 2008), these observations suggest that TMPRSS–ERG fusion alone may be insufficient for transformation from benign to malignant prostatic epithelium. However, the biological activity of alternatively spliced TMPRSS2–ERG fusion gene transcripts is pleiotropic (Wang *et al*, 2008), with the consequential effects on the emerging phenotypes of the involved neoplastic cells (initiation *vs* progression) depending on the corresponding rearrangement (Carver *et al*, 2009).

To test the hypothesis that Hsp-27 expression is a reliable predictive biomarker of clinically aggressive prostate cancer, we analysed an extensive set of tissue microarrays (TMAs) constructed from the archived diagnostic tissues obtained by the Transatlantic Prostate Group following interrogation of 6 UK Cancer Registries (Cuzick *et al*, 2006). This cohort of patients, with a detailed clinical follow-up extending over 15 years, has provided an opportunity to assess the time over which Hsp-27 expression might have predictive accuracy. As we had already utilised this cohort to confirm the predictive power of ETS-gene alterations, we were able to assess the relationship between ETS-gene status and Hsp-27 expression.

## Materials and methods

### Patient cohort

To help identify and optimise markers that may be of use in the treatment of men with prostate cancer, we recently established a retrospective cohort of men who were managed only conservatively (Cuzick *et al*, 2006; Eastham *et al*, 2008). Improving on previous studies (Chodak *et al*, 1992; Albertsen *et al*, 1995, 2005; Adolfsson *et al*, 1997; Holmberg *et al*, 2002; Johansson *et al*, 2004; Bill-Axelson *et al*, 2005), our analyses included centrally assigned Gleason scores determined by modern grading criteria and allowed comparisons with several additional clinical parameters. In agreement with Johansson *et al* (2004) and Albertsen *et al* (2005), we found Gleason score to be an important determinant of cancer-specific mortality, but baseline PSA and, to a lesser extent, stage of disease added further predictive value. Tissue microarrays were constructed from an unselected transurethral resection of the prostate specimens taken from patients who had received no initial treatment in a cohort of conservatively managed men with prostate cancer. Men who had had hormone therapy before diagnostic biopsy were also excluded, because of the influence of hormone treatment in the interpretation of Gleason grade (Cuzick *et al*, 2006).

#### Tissue micro-arrays for Hsp-27 analysis and for ETS-rearrangement FISH studies

The original haematoxylin and eosin (H&E)-stained diagnostic tissues for all cases included in this study were reviewed by a panel of three urological pathologists (DMB, CSF and VER) as previously described to confirm each diagnosis of prostate cancer and to exclude all false positives (Berney *et al*, 2007b). Of the total number of cases reviewed, 133 (7%) were reassigned a non-malignant diagnosis and thereafter excluded. The remaining 1656 cases were further reviewed to confirm and standardise the Gleason score according to conventional criteria (Deshmukh and Foster, 1997). For those 1656 cases of cancer, there was a significant reassignment in the Gleason score across a wide spectrum, yielding a more accurate predictor of prognosis than the original scores in multivariate analysis (Berney *et al*, 2007a). Following mark-up of the tissue sections to identify the predominant patterns of prostate cancer and of non-malignant glandular epithelium, TMAs were constructed in 35 × 22 × 7 mm^3^ blocks of Lamb paraffin wax using a manual tissue microarrayer (Beecher Instruments, Sun Prairie, WI, USA). Up to four cores of 600 *μ*m diameter were taken from each prostatic tissue. Reassignment of areas of cancer or non-malignant epithelium in each core was performed by histopathological examination of H&E- and p63/AMACR-stained sections that flanked the TMA slice used for FISH studies. The morphological criteria for selection of ‘normal’ and ‘malignant’ prostatic epithelium conformed to previously published definitions (Foster, 2000; Foster *et al*, 2000, 2004). FISH studies to detect *ERG* and *ETV1* gene re-arrangements were conducted as described previously (Attard *et al*, 2008a, 2008b).

#### Ethical approval

Approval for the collection of the cohort was obtained from the Northern Multi-Research Ethics Committee, followed by approval from the local ethics committee at each of the collaborating hospital trusts. This work was approved by the Clinical Research and Ethics Committee at the Royal Marsden Hospital and The Institute of Cancer Research.

#### Hsp-27 immunohistochemistry

A mouse monoclonal antibody to Hsp-27 was purchased from Leica Microsystems (NCL-Hsp-27 – Leica Microsystems Newcastle Ltd, Newcastle Upon Tyne, UK). The ADVANCE Autostainer Universal Staining System, rabbit/mouse HRP was purchased from Dako (K4068, Dako UK Ltd, Ely, Cambridge, UK). High-temperature antigen retrieval was performed using a domestic stainless-steel pressure cooker at full pressure for 3 min in 10 mM EDTA solution (pH 7.0). The Hsp-27 antibody was diluted in REAL Antibody Diluent (S2022, Dako UK Ltd) to 1 : 50. Sections of each specimen, of 4 *μ*m thickness, were cut onto amino-propyl tri-ethoxysilane-coated glass slides and dried overnight in an oven at 56 °C. For staining, slides were dewaxed with xylene, followed by rehydration through graded ethanols. Endogenous peroxidase activity was blocked by immersion in a 3% (w/v) solution of H_2_O_2_ in methanol for 12 min. Sections were then rinsed in tap water followed by deionised water. High-temperature antigen retrieval was performed, after which slides were transferred to an automatic immunostainer (Autostainer Plus, Dako UK Ltd). After equilibration in fresh TBS comprising 0.05 M Tris (pH 7.6) containing 0.12 M NaCI and 0.05% Tween-20 (TBS-T), sections were incubated with the primary antibody for 40 min at room temperature, washed with TBS-T and incubated with ADVANCE link reagent for 20 min at room temperature, washed with TBS-T and incubated with ADVANCE enzyme reagent for 30 min at room temperature. Thereafter, sections were washed before applying a solution of 3,3′-diaminobenzidine tetrahydrochloride and H_2_O_2_ (DAB+, K3468, Dako UK Ltd) for 10 min to reveal sites of antibody binding. Following a rinse in deionised water, slides were removed from the Autostainer. Nuclei were counterstained with Mayer's haematoxylin before mounting the slides in DPX. For the negative control used in each experiment, the primary antibody was replaced with Antibody Diluent. All sections were scored using CSF and ARD.

#### Analysis of Hsp-27 expression

Specimens were considered positive only if at least 5% of the epithelial cells (either normal or malignant) unequivocally expressed Hsp-27 staining. The 5% cut-off was chosen because it conforms to the International European Organization for Research and Treatment of Cancer-Gynaecological Cancer Cooperative Group recommendations (van Diest *et al*, 1997). Furthermore, this cut-off was used as the criterion to distinguish positive and negative immunohistochemical staining in our previous studies of prostate cancer (Cornford *et al*, 2000), thus ensuring consistency of criteria between studies. For each tissue section, staining was assessed as negative, weakly positive or only focally positive (low-level expression), or strongly positive (high-level expression), and scored as 0, 1, 2 or 3, respectively. For positive cores, the cellular distribution of Hsp staining in benign or malignant prostatic epithelium was assessed.

#### Statistical analyses

The primary end points for this study were time to death from prostate cancer and time to death from any cause. Univariate and multivariate analyses were performed by proportional hazard (Cox) regression analysis (Cox and Oakes, 1984). All follow-up times commenced at the point of 6 months following diagnosis, as in the previous report (Cuzick *et al*, 2006). Associations between categorical data were examined using the *χ*^2^ test and Fisher's exact test when expected cell counts were less than 5. Associations between categorical and numerical variables were assessed using analysis of variance. All *P*-values were two-sided. The following variables, determined as described previously (Cuzick *et al*, 2006), were included in the multivariate analyses: centrally reviewed Gleason score, baseline PSA (the last PSA value within 6 months of diagnosis) and age at diagnosis. Clinical stage information, which was available for 60% of the patients, was of minimal significance in this restricted series and was therefore not included.

## Results

### Hsp-27 expression in prostate tissue

**Figure 1:**
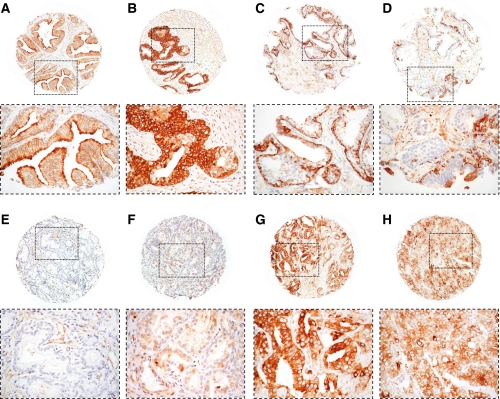
Immunohistochemical expression of Hsp-27 in TMA cores of prostatic tissues. The location of the high-magnification regions shown below each of the cores is indicated by the corresponding rectangular field. Stromal expression was not identified in any of the malignant tissues examined. No expression of Hsp-27 was identified in the nuclei although the amounts may be below the level of immunohistochemical detection. (**A**) Normal prostatic tissue in which luminal epithelial cells express Hsp-27 within the cytoplasm. Basal cells are scanty but, where present, are also stained. Stromal tissues are not stained. (**B**) Hyperplastic but not dysplastic glandular epithelium strongly expressing Hsp-27 within the cytoplasm. (**C**) Hyperplastic and mildly dysplastic epithelium in which basal cells are prominent and uniformly express Hsp-27. The overlying luminal epithelial cells are mainly negative, although a few express Hsp-27 strongly. (**D**) Hyperplastic, dysplastic and focally neoplastic intra-glandular epithelium that is predominantly negative for Hsp-27. Occasionally small foci of basal and luminal epithelial cells strongly express Hsp-27. (**E**) Moderately differentiated (Gleason 3+3) prostatic adenocarcinoma that is negative for Hsp-27 expression. (**F**) Moderately differentiated (Gleason 3+3) prostatic adenocarcinoma expressing Hsp-27 at a low level (+) in the cytoplasm of the majority of the malignant cells. (**G**) Poorly differentiated (Gleason 4+3) prostatic adenocarcinoma expression of Hsp-27 at a high (+++) level in the cytoplasm of the majority of the malignant cells. (**H**) Very poorly differentiated (Gleason 5+5) prostatic adenocarcinoma heterogeneously expressing Hsp-27 at an intermediate level (++) in the majority, but not all of the malignant cells. Magnification of all cores: × 60. The detailed fields within each of the cores are magnified at × 200.

**Figure 2:**
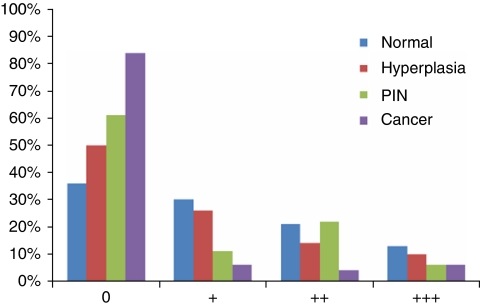
Numerical distribution of Hsp-27 expression by the epithelial cells in normal, hyperplastic, PIN and malignant tissues (cancer cores). The clinical behaviour of the malignant cells expressing Hsp-27 is shown in Figure 3. The +, ++ and +++ signs are a conventional semi-quantitative assessment of the amount of staining defined in Figure 1 legend.

Analysis of the cores of tissues contained in the 24 blocks comprising the arrays showed that 743 patients were represented on the arrays that included 1251 cancer cores, 18 PIN, 624 non-PIN hyperplasia and 792 cores with morphologically benign tissues. The distribution of cytoplasmic Hsp-27 expression within the various morphological groups ranging from benign to malignant is presented in Figure 1. In this analysis, Hsp-27 expression was identified in 64% of the cores containing morphologically unremarkable prostatic epithelia, and in 50% of the cores of hyperplastic tissues (Figure 2). Of the 18 PIN cores only 7 (39%) were positive, while 200 (16%) of the prostate cancer cores were positive. When the data for the malignant tissues were analysed with respect to individual cases, 553 patients remained in the analysis, with 441 (79.7%) of the cases being negative for Hsp-27 and 112 (20.3%) of the cases showing cytoplasmic positivity. The patients' demographics and tumour characteristics, together with details of their Hsp-27 expression, are presented in Table 1. Hsp-27 expression was associated with Gleason score (*P*=0.001), whereas no association was found with age, baseline PSA, clinical stage or extent of disease.

#### Overall effect of Hsp-27

**Figure 3:**
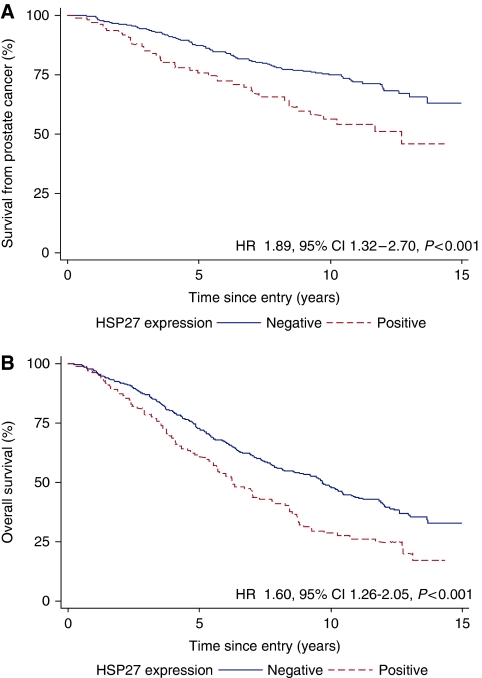
(**A**) Prostate cancer survival and (**B**) overall survival according to Hsp-27 expression.

Absence of Hsp-27 expression was associated with a better survival from prostate cancer (Figure 3) and overall survival. In the univariate analyses, Hsp-27 cytoplasmic positivity was a significant prognostic factor for poor survival from prostate cancer (HR=1.89, 95% CI=1.32–2.70, *P*<0.001) and overall survival (HR=1.60, 95% CI=1.26–2.05, *P*<0.001). In multivariate analyses that included Gleason score, extent of disease, baseline PSA and age at diagnosis, Hsp-27 cytoplasmic positivity remained an independent prognostic factor for poor cancer-specific survival (Δ*χ*^2^(1df)=4.49, *P*=0.03) and overall survival (Δ*χ*^2^(1df)=7.45, *P*=0.005).

#### Subgroup analyses

**Figure 4:**
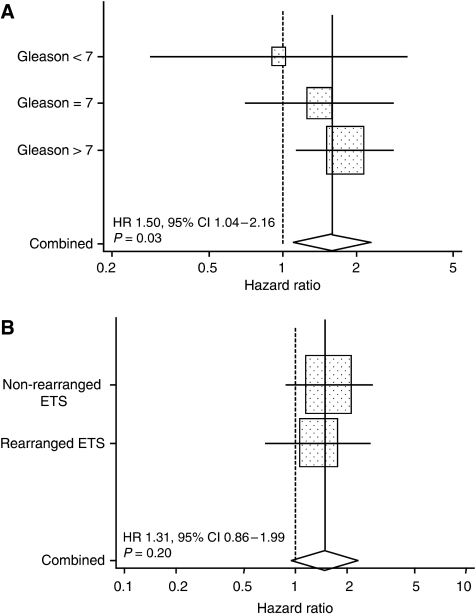
Forest plots indicating the effect of hazard ratios of Hsp-27 expression on prostate cancer survival in a Cox multivariate model by (**A**) Gleason subgroups and (**B**) ETS subgroups. The graph shows for each subgroup the hazard ratio with the 95% confidence interval and the percentage weight contributed to the meta-analysis by each subgroup is shown by the size of the corresponding rectangle. The summary hazard ratio is shown by the solid line and the middle of the diamond, the extremes of which represent the 95% confidence intervals. The dotted line shows the no effect point.

Analysis of Hsp-27 expression according to Gleason score: The independent prognostic significance of Hsp-27 cytoplasmic positivity for prostate cancer survival in a multivariate model was maintained in prostate cancers with Gleason score >7 (Δ*χ*^2^(1df)=7.08, *P*=0.03 for prostate cancer survival), but not in prostate cancers with Gleason score 7 (Δ*χ*^2^(1df)=0.43, *P*=0.50) or in prostate cancers with Gleason score <7 (Δ*χ*^2^(1df)=0.11, *P*=0.75), as shown schematically in Figure 4.

Analysis of Hsp-27 expression according to ETS-re-arrangement status: Both Hsp-27 expression and ETS (*ERG* and *ETV1*) gene status were available for 435 of the 553 patients (Table 2). When the cancers were stratified according to ETS-gene status, in multivariate analyses the presence of Hsp-27 cytoplasmic positivity failed to reach significance for prostate cancer survival in both non-rearranged ETS (Δ*χ*^2^(1df)=2.35, *P*=0.12) and rearranged ETS status (Δ*χ*^2^(1df)=0.68, *P*=0.40) groups. However, interrogation of the data (Table 2) revealed a significant difference in the distribution of Hsp-27 staining in the two groups (*χ*^2^=18.3, *P*<0.001). With increasing expression of Hsp-27 there was a progressive decline in ETS-gene rearrangement frequency (*χ*^2^(trend)=31.4, *P*<0.001), such that most of the strongly staining cancers were found in ERG non-rearranged tumours.

## Discussion

This study has shown that, at diagnosis, Hsp-27 expression is a powerful independent predictive biomarker for survival from prostate cancer (*P*<0.001) and for overall survival (*P*<0.001). Using a large cohort of men gathered from across the United Kingdom over a period of more than 15 years, Hsp-27 expression was used to accurately segregate men with poor prognosis of prostate cancer and who required active treatment from those with relatively indolent disease that could be managed conservatively, with confidence. In this study, Hsp-27 expression correlated strongly with Gleason grade (*P*=0.001), but not with baseline PSA values (*P*>0.2), despite PSA being an independent predictive variable in the original cohort of patients. The latter observation reflects the complex relationships between Hsp-27 and androgen receptor in the transactivation of PSA (Zoubeidi *et al*, 2007). The current findings also reveal that while most morphologically benign tissues strongly express Hsp-27, lower proportions of hyperplasia and PIN exhibit staining (Figure 1), suggesting that expression of the protein may be down-regulated in the *in-situ* neoplastic epithelial cells, which are the precursors of invasive prostate cancer (Foster *et al*, 2002).

There is strong evidence that Hsp-27 is an important gatekeeper of cancer cell migration and survival through differential phosphorylation and by its relative level of expression. Evidence from different cell systems indicates that Hsp-27 controls cell locomotion by a common mechanism. In fibroblasts (Hirano *et al*, 2004), endothelium, neutrophils and epithelial cells, phosphorylation and polymerisation of Hsp-27 modulates transformation of G → F actin (Tak *et al*, 2007) to generate the mechanical force required for cell motility to occur through organisation of cytoskeletal components (Doshi *et al*, 2009). In endothelial cells, Hsp-27 co-localises with and regulates assembly of actin filaments following phosphorylation by p38 MAP kinase (Guay *et al*, 1997; Landry and Huot, 1999). Thereafter, the phosphorylated and non-phosphorylated forms of Hsp-27 become differentially distributed throughout the lamellipodia, which, in the presence of fascin (Vignjevic *et al*, 2006), enable cell migration (Nagy *et al*, 2008). Non-phosphorylated Hsp-27, excluded from the leading edge of the lamellipodia, maintains short-branched actin molecules, whereas phosphorylated Hsp-27 at the base of the lamellipodia stabilises actin networks composed of long unbranched filaments (Pichon *et al*, 2004). When Hsp-27 phosphorylation is inhibited and functional interaction with actin impaired, migration dependent on F-actin polymerisation is prevented (Chen *et al*, 2009).

Enhanced levels of Hsp-27, together with its relocation from the cytoplasm to the nucleus (Wissing and Jaattela, 1996), is associated with increased resistance to apoptosis (Samali and Cotter, 1996). However, inhibition of apoptosis by Hsp-27 involves at least two parallel pathways. In the first, phosphorylated dimers of Hsp-27 interact with Daxx and block Fas-mediated apoptosis, but with no effect on Fas-induced FADD and caspase-dependent apoptosis (Charette *et al*, 2000). In the second, following interaction of phosphorylated Hsp-27 with the cytochrome *c*/Apaf-1/dATP complex, the Fas-adaptor FADD down-regulates the procaspase 9-dependent apoptotic pathway (Gibbons *et al*, 2000; Jolly and Morimoto, 2000; Charette *et al*, 2001). In the prostate, down-regulation of apoptosis is associated with elevated levels of Hsp-27, such that PC-3 and LNCaP cells become resistant to both chemical- and radiation-induced apoptosis (Gibbons *et al*, 2000; Jolly and Morimoto, 2000).

In non-malignant cells Hsp-27 is phosphorylated by MAP kinase p38 (Rouse *et al*, 1994; Huot *et al*, 1995), thus maintaining cellular homeostasis and integrity. Thereafter, Hsp-27 exists in an auto-phosphorylating signal complex together with Akt, p38 MAPK and MK2, in which Akt becomes phosphorylated on Ser^473^ by MK2 (Wu *et al*, 2007). When Hsp-27 dissociates from the complex before Akt activation, apoptosis is induced. In androgen-independent prostate cancer, lethal progression is associated with reduced apoptosis (Berges *et al*, 1995) and enhanced tumour cell proliferation (McLoughlin *et al*, 1993; Berney *et al*, 2009). Mechanistically, Hsp-27 activity is regulated by post-translational phosphorylation at serine residues Ser^15^, Ser^78^ and Ser^82^ and at theonine residue Thr^143^ (Landry *et al*, 1992). However, this process appears to be promiscuous in cancer cells, being dependent on the particular kinase activation pathway invoked and the individual cell system involved. In response to extra- or intra-cellular information, signal transducer Stat 3, controlled by its inhibitor DIAS3, alters genomic expression of Hsp-27 and facilitates phosphorylation at Ser^78^ (Song *et al*, 2004).

Comparison of the distribution and intensity of Hsp-27 expression with ETS status (Clark *et al*, 2007, 2008; Attard *et al*, 2008a, 2008b) has confirmed a statistically significant (*P*>0.001) inverse relationship between these two parameters. As the numbers of cancers containing Ets rearrangements were similar to the numbers with Ets non-rearranged in the Hsp-27-negative group, but declined rapidly with increasing expression of Hsp-27 (Table 2), it is possible that strong expression of Hsp-27 might prevent subsequent rearrangement of Ets genes if these occur subsequent to changes in Hsp-27. With respect to behavioural phenotype, strong expression of Hsp-27 and ETS-gene rearrangement are each independently associated with aggressive and rapidly lethal prostate cancer.

Not only is Hsp-27 a powerful biomarker of aggressive prostate cancer, but it is also a potential target for novel therapeutic intervention. Studies involving antisense oligonucleotides or siRNA gene knockdown showed that Hsp-27 expression promotes androgen-independent progression of prostate cancer (Rocchi *et al*, 2004, 2005). Inhibition of *HSPB1* expression modulated apoptosis and abrogated the malignant phenotype of human prostate cancer cells, thus identifying Hsp-27 as a potential therapeutic target. Separately, the biphenyl isoxasole KRIBB3 inhibits tumour cell migration by blocking protein kinase C-dependent phosphorylation of Hsp27 (Shin *et al*, 2005) to induce mitotic arrest and to enhance apoptosis (Shin *et al*, 2008). Recently, it has been shown that pyrrolo-pyrimidones, a novel class of p38 MAPK/MAPK-activated protein kinase 2 (MK2) inhibitors, inhibit phosphorylation of Hsp-27, its downstream target (Schlapbach *et al*, 2008), and that inhibition of Hsp-27 phosphorylation at Ser^78^ and Ser^82^ by the MAPKAP kinase MK5 prevents the F-actin reorganisation that is necessary for cell migration (Kostenko *et al*, 2009). Not only is the MAPKAPK2/Hsp-27 pathway a promising potential target for therapeutic intervention, but the isoflavone genistein, an oestrogen analog and candidate chemotherapeutic agent, inhibits cell migration by blocking activation of this pathway (Xu and Bergan, 2006), emphasising the validity of this proposed therapeutic approach.

This study analysing the largest cohort yet reported of conservatively managed patients with prostate cancer for more than 15 years has shown the accuracy of Hsp-27 expression as a prognostic biomarker of aggressive disease at initial diagnosis. Further, the analysis indicates two divergent phenotypes of lethal prostate cancer involving either ETS rearrangement or high Hsp-27 expression. These findings are of biological interest not only for studying the initiation and progression of prostate cancer but also for clinically identifying men who require immediate aggressive management to control potentially lethal disease. As this is a relatively small subset (∼25–35%) of men diagnosed with prostate cancer, use of this biomarker provides the biological rationale to focus the available resources on actively treating this high-risk group, while allowing the majority of prostate cancer patients (∼65–75%) to be managed conservatively.

## Figures and Tables

**Table 1:** Relationship of Hsp-27 level with demographics and tumour characteristics

		**Hsp-27 expression**		
**Variable**	**Total (*n*=553)**	**Negative (*n*=441)**	**Positive (*n*=112)**	***P*-value**
Mean age±s.d. (years)	69±5	69±5	70±5	0.43
Mean follow-up±s.d. (months)	95±49	99±48	82±47	<0.001
				
*Early hormone management*				0.21
Yes	119	90 (76%)	29 (24%)	
No	434	351 (81%)	83 (19%)	
				
*Gleason score*				0.001
<7	244	212 (87%)	32 (13%)	
=7	158	120 (76%)	38 (24%)	
>7	151	109 (72%)	42 (28%)	
				
*Clinical stage* a				0.78
T1	144	110 (76%)	34 (24%)	
T2	134	107 (80%)	27(20%)	
T3	69	52 (77%)	15 (23%)	
Unknown	206	171 (83%)	35 (17%)	
				
*Baseline PSA (ng ml* ^*−1*^ *)*				0.22
⩽4	173	147 (85%)	26 (15%)	
>4–10	102	76 (75%)	26 (25%)	
>10–25	120	97 (81%)	23 (19%)	
>25–50	95	73 (77%)	22 (23%)	
>50–100	63	48 (76%)	15 (24%)	
				
*Cancer in biopsy (%)* b				0.13
⩽6	123	104 (85%)	19 (15%)	
>6–20	139	110 (79%)	29 (21%)	
>20–40	79	68 (86%)	11 (14%)	
>40–75	83	62 (75%)	21 (25%)	
>75–100	122	91 (75%)	31 (25%)	
Unknown	7	6 (86%)	1 (14%)	

Abbreviation: PSA=prostate-specific antigen.

a Relationship with Hsp-27 calculated for patients with available clinical stage.

b Relationship with Hsp-27 calculated for patients with available percentage of cancer in biopsy.

**Table 2:** Association of Hsp-27 and ETS

	**ETS status**	
**Hsp-27**	**Normal**	**Rearranged**
Negative	185 (54%)	156 (46%)
+	19 (53%)	17 (47%)
++	23 (88%)	3 (12%)
+++	32 (100%)	0 (−)

Statistical analysis: *χ*^2^(trend)=31.4, *P*<0.001; *χ*^2^ (positive *vs* negative)=18.3, *P*<0.001.
